# Race, Ethnicity, and Other Factors Predicting U.S. Parents' Support for Policies to Reduce Food and Beverage Marketing to Children and Adolescents

**DOI:** 10.1089/heq.2018.0048

**Published:** 2018-10-17

**Authors:** Frances Fleming-Milici, Jennifer L. Harris, Sai Liu

**Affiliations:** ^1^ Rudd Center for Food Policy and Obesity, University of Connecticut, Hartford, Connecticut.; ^2^ Department of Medicine, Stanford University School of Medicine, Stanford, California.

**Keywords:** food marketing, food advertising, health disparities, obesity, public health, racial minority

## Abstract

**Purpose:** Examine parents' support for policies to reduce unhealthy food and beverage marketing to children and adolescents and identify racial, ethnic, and other sociodemographic characteristics that predict support.

**Methods:** Online survey of U.S. parents (*N*=3356) with children 2–17 years of age conducted annually (2009–2012). Participants provided attitudes about food marketing to their children, including perceived negative impact and support for food marketing-related policies. Sociodemographic characteristics examined were as follows: race, ethnicity, income, gender, political orientation, and child characteristics.

**Results:** Overall, parents agreed that food marketing negatively impacts their children's eating habits (*M*=6.87±2.08 out of 10) and supported food marketing-related policies (*M*=6.73±2.37). Perceived negative impact predicted support and was highest among black and Hispanic parents. Controlling for income and age of children in the household, Hispanic and black parents expressed highest support for policies, as did women and parents who identified as liberal or moderate in political orientation. A significant interaction between parents' political orientation and race/ethnicity indicated similarly high support among all parents, except white non-Hispanic conservative parents.

**Conclusion:** These findings are encouraging for efforts to enact policies to address unhealthy food marketing to youth. High levels of support among parents suggest advocates should continue to engage parents in their efforts. Findings also suggest that families of color would welcome policies limiting unhealthy food marketing to youth in their communities. Issues of targeted marketing and disproportionate exposure to unhealthy food marketing by black and Hispanic youth may be incorporated into campaigns to address food justice and health inequities in communities of color.

## Introduction

Extensive food and beverage marketing that promotes primarily energy-dense nutrient-poor products contributes to the epidemic of poor diet and obesity among young people.^1,2^ Black and Hispanic children and adolescents are exposed to disproportionately more unhealthy food marketing in the media^3,4^ and their neighborhoods.^5–7^ Furthermore, companies often target marketing for their least healthy products, including sugar-sweetened beverages, fast food, and candy, directly to black and Hispanic youth.^3,4,8–10^ Therefore, food marketing to black and Hispanic youth may contribute to disparities in diet-related diseases affecting communities of color.^11,12^

In the United States, initiatives to address unhealthy food marketing to youth have focused primarily on industry self-regulation.^13^ Voluntary industry self-regulatory programs have promised to improve the nutritional quality of food and beverages marketed to children^14,15^; however, independent evaluations of self-regulation have demonstrated limited improvements.^1,16^ In addition, self-regulatory initiatives have not addressed targeted marketing to youth of color, and recent studies indicate a substantial increase in disparities in food advertising exposure between black and white children and adolescents.^3,4^

In response to concerns about the lack of progress in improving food marketing to youth, policymakers have proposed and enacted some local regulations that limit unhealthy food marketing. For example, Santa Clara, CA, and San Francisco have enacted nutrition standards for fast-food children's meals that come with toys.^17,18^ At the national level, food and drinks must now meet competitive food standards to be marketed in schools.^19^ In addition, advocates have begun to mobilize parents to demand additional policies to reduce unhealthy food marketing to youth,^20,21^ and some advocacy efforts specifically address unhealthy food marketing targeted to black and Hispanic youth.^22–24^

To further this growing movement, policymakers and advocates would benefit from evidence of public support for policies to reduce unhealthy food marketing to children and adolescents. A better understanding of black and Hispanic parents' attitudes toward policies that address marketing in their communities is also needed. Some researchers have suggested that community members may view such policies as restrictive or paternalistic.^25^ However, research in this area is limited, and much of it was conducted before industry self-regulatory initiatives were established.^26–29^ Furthermore, findings on differences in support by income, race, and ethnicity have been inconsistent^26,27,30^ or inconclusive due to limited sociodemographic diversity in the sample.^31^ In addition, previous cross-sectional studies have not examined how sociodemographic characteristics interact as predictors of support. Finally, the majority of research in this area has measured attitudes of U.S. adults,^27–29,31^ not parents specifically. Yet many advocates are currently working to engage parents, and parents would benefit most directly from policies to improve the health of children and adolescents.

Parents' perceptions of harm from children's exposure to unhealthy food marketing is another potentially important, but understudied, factor in predicting support for food marketing-related policies. Experts have suggested that failure to recognize the negative impact of unhealthy food marketing is a likely barrier to support of policies.^32^ Just one study has included perceived negative impact as a predictor of support for policies, and the researchers found it to be a strong predictor.^30^ Therefore, additional research is needed to better understand relative support for policies to address unhealthy food marketing to children among parents, including parents of color and others whose children may be disproportionately impacted.

In this study, we surveyed parents of children and adolescents to identify predictors of support for a variety of policies to address food marketing to youth. The survey was conducted annually from 2009 to 2012. We assessed differences by sociodemographic characteristics, including race, ethnicity, household income, political orientation, and gender; characteristics of children in the household (age and weight status); interactions between demographic variables; and changes in support over time. We also measured parents' perceptions that food marketing negatively impacts their children's eating habits.

## Methods

Data were collected as part of a larger annual survey that measured parents' attitudes about healthy eating for their children, perceived influence of food marketing and other factors affecting their children's eating habits, and support for a variety of policies to promote healthy eating in children and adolescents.^33^ The university's human subjects committee determined the study to be exempt. Cognitive testing demonstrated validity of survey items, and consistent findings across 4 years demonstrated good reliability.^34^

The total sample across all 4 years included 3356 participants. All participants were parents with children 2- to 17-years-old living at home, screened for a minimum annual household income of $15,000 and for primary or shared responsibility for household food and beverage choices. Recruitment occurred through an online survey panel maintained by Survey Sampling International (SSI; surveysampling.com). SSI recruits panel members through thousands of websites to obtain a diverse sample of the online population. Participants accessed the survey through an e-mail link. It was conducted during June–July of 2009, 2010, 2011, and 2012. Sampling procedures, sample size, and data collection period remained consistent over the 4 years. Each year, an initial sample of ∼600 parents was recruited, augmented by at least 100 additional black and Hispanic parents each to ensure adequate sample sizes for comparison by race and ethnicity. Overall participation rates were 81% in 2009, 78% in 2010, 86% in 2011, and 80% in 2012.

### Measures

Participants answered questions about children living in their household, including age, gender, height, and weight. They also indicated on a scale of 1–10 (1=definitely would oppose/strongly disagree and 10=definitely would support/strongly agree) their support for nine potential policies to reduce unhealthy food marketing to youth and their agreement with thirteen items assessing perceptions of negative impact of food marketing on their children's eating habits (Table 1). These policies were adapted from potential federal and state policy options examined by previous researchers^35^ and include policies recently proposed by public health experts and advocates.^20,21,36,37^ Perceptions about the negative impact of food marketing on children's eating habits were adapted from previous research.^30^ In addition, participants indicated their own gender, race/ethnicity, and household income in the previous year and provided their political orientation on a scale of 1–7 (1=strongly liberal, 4=middle-of-the-road, and 7=strongly conservative). The full survey is available online.^33^

**Table 1.** **Items Measuring Parents' Support for Policies to Reduce Unhealthy Food Marketing and Perceived Negative Impact of Food Marketing**

Items	*M* (1–10)	95% CI
Support for policies to reduce unhealthy food marketing^a^		
Require children's TV programs to show children being physically active and eating healthy food	7.31	7.22–7.41
Require children's media companies to fund public service announcements for fruits and vegetables on TV	7.06	6.97–7.76
Require children's media companies to fund an equal amount of advertising for healthy and unhealthy foods	6.92	6.82–7.01
Allow only healthy food advertising on TV programs targeted to children younger than 12 years	6.75	6.65–6.85
Do not allow any advertising on TV programs targeted to children younger than 8 years	6.21	6.11–6.32
Allow only healthy food advertising on TV programs targeted to youth younger than 18 years	6.44	6.34–6.54
Allow cartoon characters only on packages for healthy foods	6.35	6.25–6.45
Allow only healthy foods and beverages in school vending machines	7.31	7.22–7.41
Do not allow games or other child-oriented features on unhealthy food websites	6.21	6.11–6.31
Scale average	6.73	6.65–6.81
Perceived negative impact of food marketing^b^		
Encourages children to ask parents for the advertised foods and beverages	7.70	7.62–7.78
Affects everyone, not just children	7.72	7.63–7.80
Increases preferences for the types of foods advertised	7.19	7.11–7.28
Promotes unhealthy foods	6.93	6.84–7.02
Encourages snacking between meals	6.88	6.79–6.97
Encourages unhealthy snacking	6.89	6.80–6.98
Leads to food cravings	6.93	6.84–7.02
Creates eating habits that stick with you for life	6.87	6.78–6.96
Affects children the most	6.86	6.77–6.95
Makes parents' jobs harder	6.52	6.42–6.61
Causes children to eat more	6.41	6.31–6.50
Encourages large portions	6.25	6.15–6.35
Affects the products you choose to buy for your children	6.20	6.11–6.30
Scale average	6.87	6.80–6.94

^a^ Response to the following question: “Below is a list of actions that are either currently being taken or could be taken to promote healthy eating habits and physical activity to your children. Using the scale below, please indicate how much you would support each of the following actions” with response options ranging from 1 (definitely would oppose) to 10 (definitely would support) Cronbach's α=0.94.

^b^ Response to the following question: “Using the scale below, please indicate how much you agree with the following statements about food and beverage marketing and advertising to your children” with response options ranging from 1 (strongly disagree) to 10 (strongly agree) Cronbach's α=0.95.

CI, confidence interval; M, mean.

### Data analysis

Responses were combined to create a scale of support for policies to reduce unhealthy food marketing and a scale for perceived negative impact of food marketing. Both scales showed excellent internal consistency (Cronbach's α=0.94 and 0.95, respectively; Table 1). Children's weight status was calculated using parents' reports of children's gender, age, height, and weight. Children whose body mass index (BMI)-for-age fell between the 85th and 95th percentile were classified as having overweight, and those with a BMI-for-age above the 95th percentile were classified as having obesity, according to U.S. Centers for Disease Control and Prevention growth charts (www.cdc.gov/growthcharts). Parents with one or more child with overweight or obesity were identified. Parents were grouped according to age of oldest child (2–5, 6–11, and 12–17 years). Political orientation was coded as liberal (1–3), moderate (4), and conservative (5–7). Lower ($15,000–$40,000), moderate ($40,001–$75,000), and higher income (>$75,000) were coded.

One-way analysis of variance and *t*-tests compared scale means between sociodemographic groups and changes by year. Significance of multiple comparisons was adjusted using Tukey's *post hoc* tests. Sociodemographic variables, perceived negative impact, year of survey, and all two-way interactions between sociodemographic groups were included in an initial linear regression model to predict support for policies to reduce food marketing to youth. To assess the contribution of sociodemographic predictors of support alone, perceived negative impact was removed through backward elimination. The final model included only sociodemographic variables associated with the outcome and two-way interaction terms at *p*<0.05 significance. All analyses were conducted using SAS (version 9.2; SAS Institute, Inc., Cary, NC).

## Results

Two-thirds of participants were 25–49 years of age and approximately two-thirds were female. The sample was highly diverse (Table 2). Almost one-half of participants were black, Hispanic, or mixed/other race/ethnicity. More than one-third lived in households with incomes of $40,000 or less, while one-quarter lived in households with incomes of $75,000 or higher. Approximately one-half of participants self-identified as moderate in political orientation, while conservative parents outnumbered liberal parents (33% vs. 20%). Forty-five percent of parents had at least one child with overweight or obesity, and one-half had at least one teenage child.

**Table 2.** **Sample Characteristics and Differences in Scale Responses Between Sociodemographic Groups**

	Sample size	Percentage	Support for policies to reduce food marketing		Perceived negative impact of food marketing	
	*n*	(%)	M (1–10)	95% CI	M (1–10)	95% CI
**Total sample**	3,356	100%	6.73	(6.65–6.81)	6.87	(6.80–6.94)
**Gender**						
Female	2,281	68%	6.90^a^	(6.81–7.00)	6.91	(6.83–7.00)
Male	1,075	32%	6.36^b^	(6.21–6.52)	6.79	(6.67–6.92)
**Race/ethnicity**						
White non-Hispanic	1,711	51%	6.33^a^	(6.21–6.45)	6.69^a^	(6.58–6.79)
Black	687	20%	7.10^b^	(6.95–7.26)	7.03^b^	(6.88–7.17)
Hispanic	810	24%	7.19^b^	(7.05–7.34)	7.10^b^	(6.96–7.24)
Mixed/others^*^	148	4%	7.06	(6.71–7.42)	7.11	(6.80–7.42)
**Household income**						
$15,000–$40,000	1,312	39%	6.84^a^	(6.71–6.96)	6.85	(6.74–6.97)
$40,001–$75,000	1,222	36%	6.74^ab^	(6.61–6.88)	6.94	(6.83–7.06)
More than $75,000	822	24%	6.57^b^	(6.37–6.70)	6.80	(6.66–6.95)
**Political orientation**						
Liberal	655	20%	6.93^a^	(6.76–7.11)	7.22^a^	(7.07–7.36)
Moderate	1,608	48%	6.88^a^	(6.78–6.99)	6.83^b^	(6.73–6.93)
Conservative	1,093	33%	6.38^b^	(6.22–6.54)	6.74^b^	(6.60–6.87)
**Any child with overweight/obesity**						
Yes	1,370	45%	6.84^a^	(6.72–6.96)	7.00^a^	(6.89–7.11)
No	1,687	55%	6.59^a^	(6.47–6.71)	6.75^b^	(6.65–6.85)
**Age of oldest child**						
2–5 years old	631	19%	6.89^a^	(6.72–7.07)	6.90	(6.74–7.06)
6–11 years old	1,001	30%	6.85^a^	(6.71–6.99)	6.90	(6.77–7.02)
12–17 years old	1,724	51%	6.60^b^	(6.49–6.72)	6.85	(6.75–6.95)
**Year of survey**						
2009	859	26%	6.66	(6.50–6.82)	6.79	(6.65–6.92)
2010	797	24%	6.64	(6.47–6.80)	6.83	(6.68–6.98)
2011	798	24%	6.74	(6.57–6.91)	6.91	(6.76–7.05)
2012	902	27%	6.87	(6.72–7.02)	6.96	(6.83–7.10)

Note: Within each row and category, only those means that do not share a common superscript differ significantly at *p* ≤ 05. Means that share a common superscript (or with no superscript) do not differ significantly from each other.

^*^ Excluded from race/ethnicity analysis.

Table 2 also presents scale means. On average, all demographic groups supported food marketing-related policies (*M*=6.73 out of 10). In the bivariate analysis, black and Hispanic parents were significantly more supportive than white non-Hispanic parents, *F*(2,3045)=32.19, *p*<0.001, and parents with household incomes of $40,000 or less expressed greater support than did parents with incomes >$75,000, *F*(2,3045)=3.39, *p*=0.03. Women were more supportive than men, *t*(3354)=6.15, *p*<0.001. Politically conservative parents were less supportive than liberal and moderate parents *F*(2,3045)=19.40, *p*<0.001. Having a child with overweight/obesity also predicted support, *t*(3046)=−2.85, *p*=0.005; and parents of teenagers were less supportive than parents with younger children only, *F*(2,3045)=3.11, *p*=0.04. Overall, mean support did not change from 2009 to 2012, *p*=0.24.

As with policy support, on average, parents agreed that food marketing negatively impacts their children's eating habits (*M*=6.87 out of 10), with both black and Hispanic parents perceiving greater negative impact than white non-Hispanic parents, *F*(2,3045)=9.76, *p*<0.001. Liberal parents perceived a greater negative impact than did moderate and conservative parents, *F*(2,3045)=12.99, *p*<0.001; and parents with at least one child with overweight/obesity also perceived a greater negative impact than did other parents, *t*(3046)=3.29, *p*=0.001. However, there were no significant differences in perceived negative impact for mothers versus fathers, or by household income or age of oldest child. Similar to policy support, perceived negative impact did not change from 2009 to 2012.

**FIG. 1.**
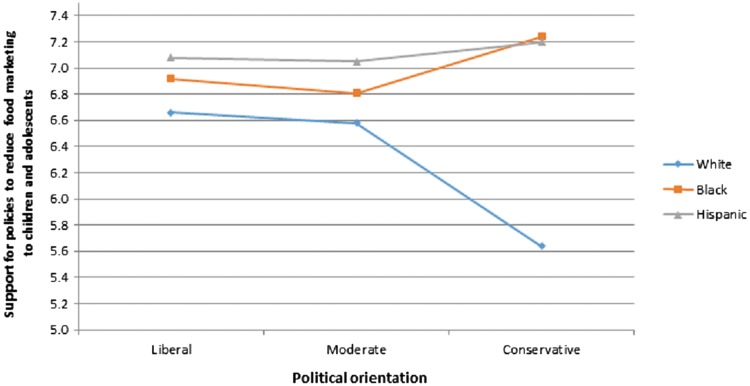
Interaction between race/ethnicity and political orientation in parents' support for policies to reduce food marketing to children and adolescents.

Table 3 presents the models predicting support for policies to reduce unhealthy food marketing to children. As expected, perceived negative impact of food marketing on their children was strongly associated with parents' support for food marketing-related policies. Independent of perceived negative impact, mothers, black and Hispanic parents, and liberal and moderate parents expressed significantly higher support. However, controlling for perceived negative impact and other sociodemographic characteristics, children's weight status, age of children in the household, and household income no longer predicted policy support. The only significant two-way interaction found was between parents' race/ethnicity and political orientation, such that support was similarly high for all parents (*M*=6.64–7.24 out of 10) with the exception of white non-Hispanic parents who identified as politically conservative (Fig. 1). Although these parents expressed significantly lower support than other parents, on average, they did support policies (*M*=5.65 out of 10). After removing perceived negative impact from the model, parents with at least one child with overweight/obesity also expressed significantly higher support for policies to reduce unhealthy food marketing to children, in addition to black and Hispanic parents, liberal and moderate parents, and mothers.

**Table 3.** Stepwise Generalized Linear Regression for Models to Predict Support for Policies to Reduce Food Marketing to Youth

Variables	Full model			Final model^a^		
	Estimate (B)	Standard error (SE)	Standardized estimate (β)	Estimate (B)	Standard error (SE)	Standardized estimate (β)
Constant	1.00	0.15		5.57	0.10	N/A
Perceived negative impact of food marketing	0.73	0.02	0.64^*^			
Year of survey						
(2009 as reference)						
2010	−0.04	0.09	0.00			
2011	0.11	0.09	0.02			
2012	0.12	0.09	0.02			
Age of oldest child						
(12–17 years old as reference)						
6–11 years old	0.13	0.07	0.03			
2–5 years old	0.18	0.09	0.03			
Household income						
($15,000–$40,000 as reference)						
$40,001–$75,000	−0.14	0.07	−0.03			
>$75,000	−0.13	0.08	−0.02			
Parents' gender						
(Male as reference)						
Female	0.2	0.03	0.08^*^	0.25	0.05	0.10^*^
Race/ethnicity						
(White non-Hispanic as reference)						
Black	0.91	0.16	0.15^*^	1.59	0.21	0.27^*^
Hispanic	0.94	0.14	0.17^*^	1.56	0.19	0.28^*^
Political orientation						
(Conservative as reference)						
Moderate	0.58	0.10	0.12^*^	0.92	0.13	0.19^*^
Liberal	0.49	0.13	0.06^*^	0.99	0.16	0.16^*^
Child with overweight/obesity						
(No as reference)						
Yes	0.00	0.06	0.00	0.16	0.09	0.03^**^
Race/ethnicity by political orientation	0.18	0.05	0.17^*^	0.25	0.06	0.24^*^

β, standardized beta; B, nonstandardized coefficient; SE, standardized coefficient.

^*^ *p*<0.001.

^**^ *p*<0.05.

^a^ Final model excludes perceived negative impact of food marketing and nonsignificant variables.

## Discussion

This research demonstrates that, on average, parents across all sociodemographic groups examined support policies to reduce unhealthy food marketing to children. However, the research also highlights differences between some groups. For example, men and parents who identify as politically conservative were less supportive of policies to reduce food marketing to children and adolescents overall, while both black and Hispanic parents, regardless of political orientation, were more supportive. These findings also confirm previous research demonstrating that perceived negative impact of food marketing on their children is an important predictor of parents' support for policies to reduce food marketing to youth.^30^ Of note, support for food-marketing policies did not change from 2009 to 2012, despite a period of food and beverage industry efforts to promote their progress in improving food marketing to children.^14,38^

These research findings can help advocates identify key constituents for grassroots movements and coalitions aimed at enacting policies to reduce unhealthy food marketing to youth, as well as provide policymakers with evidence of potential support for such policies.

Notably, beliefs that food marketing negatively impacts their children and support for policies to reduce their children's exposure to such marketing were highest among black and Hispanic parents, independent of income, political orientation, and their children's weight status. This finding suggests significant opportunities for child health advocates to enlist communities of color in addressing unhealthy food marketing to youth. It appears that advocates would find broad support from black and Hispanic community members and an opportunity to expand grassroots campaigns to reduce the marketing of nutrient-poor foods to black and Hispanic children and promote such efforts as issues of social justice.^22–24^ Campaign messages that focused on targeted marketing and disproportionate exposure to marketing of sugary drinks by black and Hispanic youth have been integral to the successful enactment of soda tax legislation in U.S. municipalities.^39,40^

This research also demonstrates that parents' beliefs that food marketing negatively impacts their children are highly associated with their support for policies to reduce unhealthy food marketing. This finding provides guidance for designing policy campaign messages. The study design does not prove a causal relationship between perceived negative impact of food marketing and support for policies. However, communication campaigns to increase parents' awareness of unhealthy food marketing and understanding of how food marketing negatively impacts their children may increase support for policies and other actions to reduce their children's food marketing exposure.

These findings also highlight the need for the public health community to better understand the concerns of white non-Hispanic parents with a conservative political orientation and utilize alternative messages to address those concerns. Among politically conservative American adults, individual responsibility rather than environmental factors (i.e., food marketing to children)^26,27^ shapes attitudes regarding the causes of obesity^41^; therefore, messages that focus on how food marketing undermines parents' authorities over decisions about what to feed their children might increase support among these parents. However, it is important to note that, while these parents were less supportive overall than other parents, on average, they also supported policies to reduce unhealthy food marketing to children. Despite substantial opposition from the food and beverage industry to policy efforts to reduce unhealthy food marketing to children that suggest otherwise,^42^ this research indicates that the majority of parents would likely welcome policies to reduce unhealthy food marketing and support parents' efforts to raise healthy children.

This research has limitations. The sample is not representative of the total or voting U.S. population. However, recruitment procedures specifically oversampled black and Hispanic parents to enable comparisons by race/ethnicity. Researchers also screened for parents who had the responsibility for decisions about feeding their children, which may have resulted in a higher proportion of female respondents and parents with greater first-hand experience with the impact of unhealthy food marketing on children. In addition, using an internet panel for data collection excluded parents without computer access or the time or willingness to complete the survey. However, the findings of general support for policies to reduce unhealthy food marketing to youth, demographic differences in support, and the contribution of various predictors were generally consistent with results from previous research.^26,27,29–31^ Self-reported data presented another limitation common to all attitude and policy support research. Further, parent-reported weight and height for children are not as accurate as anthropometric measurement, although they have been shown to accurately classify BMI status of children.^43^ Finally, the cross-sectional data cannot prove causal effects or direction of causation.

## Conclusion

This research demonstrates that parents, especially black and Hispanic parents, believe that food marketing negatively impacts their children and widely support policy proposals to reduce unhealthy food marketing to youth. Furthermore, parents who likely have the most direct experience with the negative impact of unhealthy food marketing, including parents of children with overweight or obesity, as well as black and Hispanic parents, were most supportive. Even though white non-Hispanic conservative parents were less supportive than others, on average, they too supported such policies. These findings should be encouraging for policymakers and advocates who care about children's health. They provide evidence of broad support among parents for legislation and regulatory options to reduce high levels of unhealthy food marketing targeted to young children and adolescents.

## Health Equity Implications

These findings suggest that advocates will find support from black and Hispanic community members in their efforts to reduce the marketing of nutrient-poor foods to black and Hispanic children and adolescents. Targeted marketing and disproportionate exposure to unhealthy food marketing by black and Hispanic youth may be incorporated into social justice campaigns that frame unhealthy food marketing as a health equity issue.

## Abbreviations Used

BMI: body mass index
CI: confidence interval
SSI: Survey Sampling International
